# First person – Aikta Sharma

**DOI:** 10.1242/dmm.048968

**Published:** 2021-03-17

## Abstract

First Person is a series of interviews with the first authors of a selection of papers published in Disease Models & Mechanisms, helping early-career researchers promote themselves alongside their papers. Aikta Sharma is first author on ‘Multiscale molecular profiling of pathological bone resolves sexually dimorphic control of extracellular matrix composition’, published in DMM. Aikta is a PhD student in the lab of Dr Claire E. Clarkin at the University of Southampton, Southampton, UK, investigating the role of osteoblast-derived vascular endothelial growth factor in bone matrix formation.


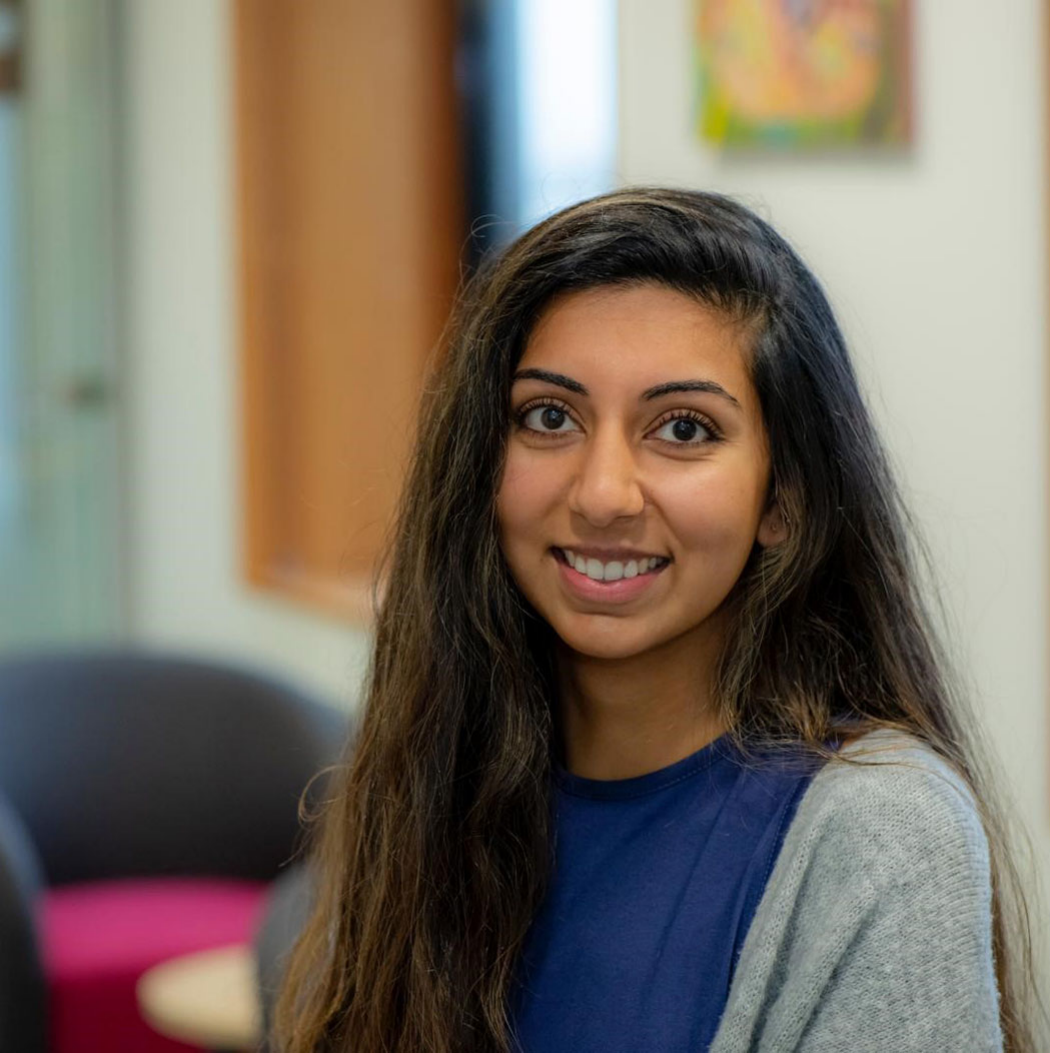


**Aikta Sharma**

**How would you explain the main findings of your paper to non-scientific family and friends?**

The development and maintenance of a healthy skeleton relies on the communication between the networks of cells that are involved in its formation. Two cell types that are key in this process are the bone-forming osteoblasts and the blood vessel-lining vascular endothelial cells. Communication between these cells occurs following vascular endothelial growth factor (VEGF) release. Reductions in circulating VEGF in the blood is seen in bone diseases such as osteoporosis, which is characterised by brittle and porous bone and a defective blood supply; therefore, keeping blood vessels healthy may provide a new way to treat the disease. In bone, the osteoblasts are considered to be the main source of VEGF. Therefore, changes in bone quality and strength in disease are considered to occur as a direct consequence of the altered ability of osteoblasts to form and arrange the bone components; namely, collagen and calcium-containing mineral crystals. Until now, these effects have been technically challenging to study as the bone tissue itself is exceptionally dense, with many of the bone cells and vessel cells encapsulated within it. In our study, using a mouse model in which *Vegf* expression was deleted in mature osteoblasts, we characterised how the loss of VEGF impacts the bone components and how they are arranged. To do this, we used novel and label-free technologies such as Raman spectroscopy, a chemical fingerprinting technique that can provide information on the quality of the bone and its composition, with second-harmonic generation microscopy, which was used to study the arrangement of collagen fibres. Our results suggest that osteoblast-derived VEGF is primarily involved in the formation and arrangement of the collagen fibres, which could be responsible for the vessel phenotype seen in this model. These effects were additionally observed to be sex dependent, with many disadvantageous changes occurring exclusively in males, making them more susceptible to fracture. Overall, our study demonstrates the utility of such novel methodologies that enable the assessment of microscopic changes that underlie common bone diseases. Such disease-related signatures could be used in accelerating the diagnosis of degenerative bone diseases in clinical settings in the future.

“[…] disease-related signatures could be used in accelerating the diagnosis of degenerative bone diseases in clinical settings in the future.”

**What are the potential implications of these results for your field of research?**

These results highlight the need to consider males and females as separate entities. Many studies utilizing mouse models perform whole studies on groups consisting of only male mice or only female mice. Where studies involving primary cell work are being conducted, the derived cells are often pooled from litters containing male and female pups, resulting in the immediate loss of any non-hormonal sex differences. If mouse models are being used to assess the mechanisms underlying pathology, bias when choosing the sex of the mice that will be used in the study may translate in the identification of non-universal biomarkers, leading to more generic therapies that may not efficiently treat a given disease. In our study, we screened a large number of genes in male and female *Vegf-*deficient osteoblasts to understand the mechanism underlying the matrix phenotype, several of which were differentially impacted by the *Vegf* loss. As sexual dimorphism in a number of diseases is now being documented, characterisation of the origins of such diseases and how they present differently in males and females may be a gateway in which scientific research can revolutionise modern medicine.

**What are the main advantages and drawbacks of the model system you have used as it relates to the disease you are investigating?**

The main advantage of using this mouse model is that we can dissect away the contributors that underlie the phenotypic defects. Currently, employing label-free and non-destructive imaging techniques alongside chemical fingerprinting is challenging in humans due to limited tissue penetration and patient sample access, heterogeneity between samples, including site of tissue removal, and differences in patient age, sex and medical history. Many of these aspects can be overcome by using mouse models, which in our study has allowed us to uniquely characterise a ‘signature’ that encompasses multiple components of bone pathology from the macroscale to nanoscale level. One of the biggest drawbacks of using mouse models to study human diseases is the anatomical differences in the long bones. Comparative in-depth studies using both mouse and human bone are pivotal for enabling the translation of findings between species, allowing common disease markers to be identified. The field of biomedical optics, however, is advancing, as fibre-optic based probes are now being used to study tissue pathology in a minimally invasive manner in a variety of organisms. I hope to see some of these modernised systems being used to diagnose both bone and a variety of other human diseases in clinical settings in the years to come.

**What has surprised you the most while conducting your research?**

My current research group has been working with this mouse model for over 5 years and, despite how much work has gone into characterizing the effects of the *Vegf* loss, we always find something new whenever we revisit old samples. Our previous work on this model showed that males exhibit most of the detrimental effects, which is conserved from the tissue to cell level. What I found most fascinating was that through the various platforms we used to carry out our analysis, such as high-resolution synchrotron computed tomography to Raman spectroscopy and second-harmonic generation microscopy, there are just so many components that are invisible to the naked eye that together translate into the defects that we have reported. Whether or not the effects on the vasculature occurs primary to the matrix phenotype is still unclear – it is similar to the question of “what comes first, the chicken or the egg?”.

**Figure:**
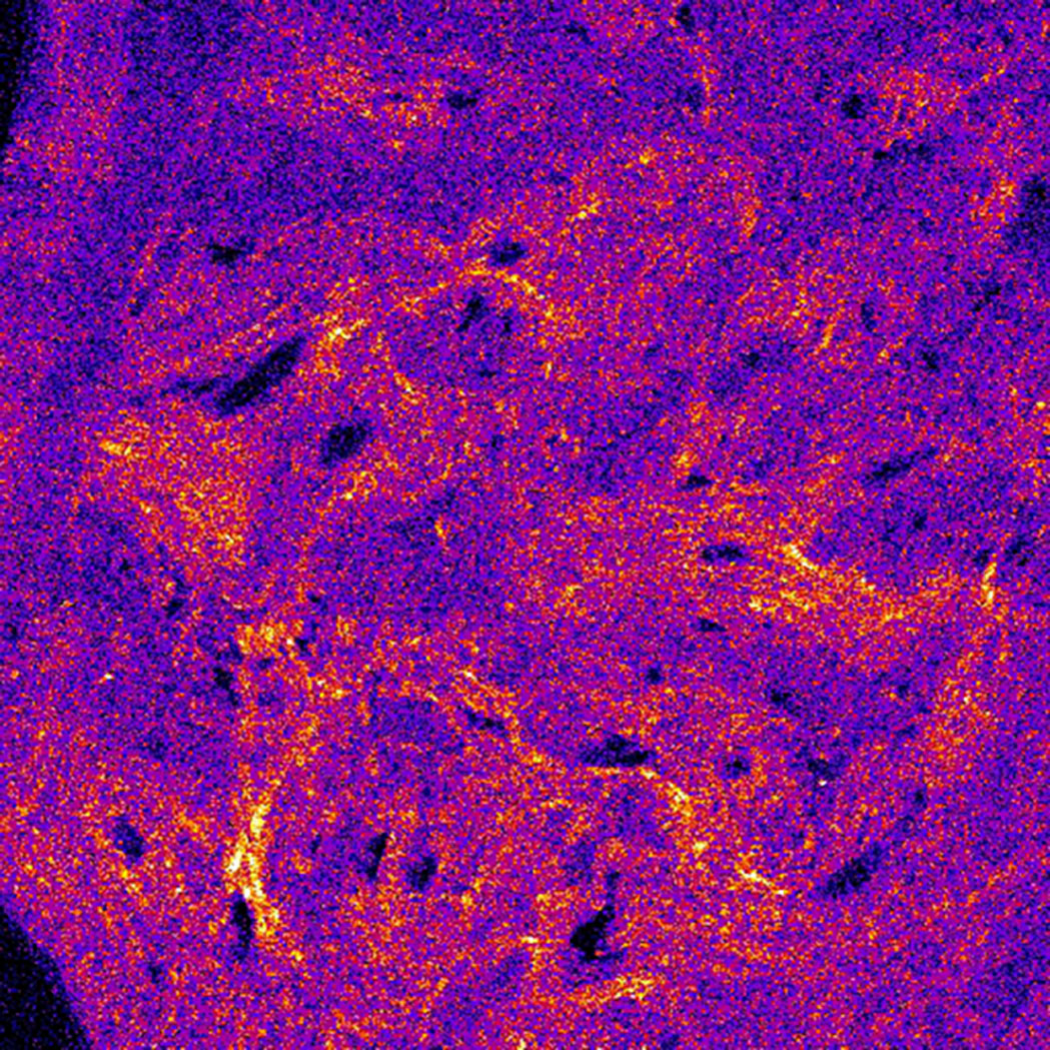
**Label-free second-harmonic generation image of disorganised collagen fibres in the bone cortex of a male conditional *Vegf* knockout mouse.**

**Describe what you think is the most significant challenge impacting your research at this time and how will this be addressed over the next 10 years?**

The biggest obstruction to research at the moment is the changes that we have had to make to our lifestyles as a result of the COVID-19 pandemic. Almost all aspects of ‘normal’ working life have changed for researchers across the globe. As a final year PhD student, it has limited the immediate outlook of my project as I have had to change the direction of my research so that the goals are achievable in a reasonable timeframe. Unfortunately, time is not on our side; this will inevitably have long-lasting effects on our research communities, from motivation and productivity to animal work and system development. Being able to adapt to this new normal is easier said than done, and it may take more time until we are comfortable enough or in a position to go back to producing the same level of output to advance science as before the pandemic.

“Networking with other early-career and senior researchers is essential in academia and beyond.”

**What changes do you think could improve the professional lives of early-career scientists?**

Networking with other early-career and senior researchers is essential in academia and beyond. The movement to virtual scientific meetings has made the science much more accessible. Late last year, I presented my research in the annual Australia and New Zealand Bone and Mineral Society meeting – I would not have been able to attend the meeting in-person due to travelling costs and registration fees. Every researcher has the opportunity to showcase their work both nationally and internationally. Our reach may also be the largest it ever will be; the audiences are bigger so we can promote our scientific vision to so many others in the field. Hearing about what other PhD students and early-career researchers are researching has always been important in being involved and part of the global research community. Participation in any conference – big or small, formal or informal – will help us in the interim until in-person conferences and travel is possible.

**What's next for you?**

Once I have completed my PhD, I will be continuing my research training as a postdoc in my current lab group.
